# Competing endogenous RNA network analysis explores the key lncRNAs, miRNAs, and mRNAs in type 1 diabetes

**DOI:** 10.1186/s12920-021-00877-3

**Published:** 2021-02-01

**Authors:** Chang Li, Bo Wei, Jianyu Zhao

**Affiliations:** 1 Departments of VIP Unit, China-Japan Union Hospital of Jilin University, Changchun, 130033 Jilin China; 2 Departments of Neurosurgery, China-Japan Union Hospital of Jilin University, Changchun, 130033 Jilin China; 3 Department of Endocrinology, China-Japan Union Hospital of Jilin University, 126 Xiantai Street, Changchun, 130033 Jilin China

**Keywords:** Type 1 diabetes, Long non-coding RNA, mRNA, microRNA, Differential expression analysis, Interaction network

## Abstract

**Background:**

Type 1 diabetes (T1D, named insulin-dependent diabetes) has a relatively rapid onset and significantly decreases life expectancy. This study is conducted to reveal the long non-coding RNA (lncRNA)-microRNA (miRNA)-mRNA regulatory axises implicated in T1D.

**Methods:**

The gene expression profile under GSE55100 (GPL570 and GPL8786 datasets; including 12 T1D samples and 10 normal samples for each dataset) was extracted from Gene Expression Omnibus database. Using limma package, the differentially expressed mRNAs (DE-mRNAs), miRNAs (DE-miRNAs), and lncRNAs (DE-lncRNAs) between T1D and normal samples were analyzed. For the DE-mRNAs, the functional terms were enriched by DAVID tool, and the significant pathways were enriched using gene set enrichment analysis. The interactions among DE-lncRNAs, DE-miRNAs and DE-mRNAs were predicted using mirwalk and starbase. The lncRNA-miRNA-mRNA interaction network analysis was visualized by Cytoscape. The key genes in the interaction network were verified by quantitatively real-time PCR.

**Results:**

In comparison to normal samples, 236 DE-mRNAs, 184 DE-lncRNAs, and 45 DE-miRNAs in T1D samples were identified. For the 236 DE-mRNAs, 16 Gene Ontology (GO)_biological process (BP) terms, four GO_cellular component (CC) terms, and 57 significant pathways were enriched. A network involving 36 DE-mRNAs, 8 DE- lncRNAs, and 15 DE-miRNAs was built, such as *TRG-AS1*—miR-23b/miR-423—*PPM1L* and *GAS5*—miR-320a/miR-23b/miR-423—*SERPINA1* regulatory axises. Quantitatively real-time PCR successfully validated the expression levels of *TRG-AS1*- miR-23b -*PPM1L* and *GAS5*-miR-320a- *SERPINA1*.

**Conclusion:**

*TRG-AS1*—miR-23b—*PPM1L* and *GAS5*—miR-320a—*SERPINA1* regulatory axises might impact the pathogenesis of T1D.

## Background

As a metabolic disorder characterized by hyperglycemia, diabetes is induced by secretion deficiency or impaired biological function of insulin [1, 2]. Diabetes can result in impairment and dysfunction of multiple tissues, including heart, blood vessels, eyes, nerves, and kidneys [3–5]. According to the WHO classification of diabetes mellitus in 2019, diabetes are classified as type 1 diabetes (T1D), type 2 diabetes (T2D), hybrid forms of diabetes, other specific types, such as monogenic diabetes, unclassified diabetes and hyperglyacemia first detected during pregnancy [6, 7]. The symptoms of T1D mainly include thirst, polydipsia, polyuria, polyphagia, fatigue, and rapid weight loss [8].An annual 3%—4% increase in the incidence of T1D in childhood was estimated in several developed countries [9]. Besides, T1D decreases approximately 11–13 years of life expectancy in developed countries, and even more time in developing countries [10]. Quality of life of T1D patients was significantly decreased than general population. Therefore, investigation in the pathogenesis of T1D is warranted to improve the prognosis of T1D patients.

Considerable efforts have been made on the molecular mechanism of T1D during the past decades. The roles of non-coding RNAs, including microRNA (miRNA), long non-coding RNAs (lncRNAs) in the regulation of T1D have only recently been recognized [11]. Through reducing C–C motif chemokine receptor 2 expression, miR-125a-5p limits the migration and function of regulatory T cells (Tregs) in the pancreas of T1D patients [12]. Activity enhancement of miR-181a promotes the expression of nuclear factor of activated T cells 5 (*NFAT5*) while suppresses the induction of forkhead box P3 + Tregs; therefore, miR-181a/*NFAT5* axis may provide targets for limiting islet autoimmunity in T1D patients [13, 14]. The sera level of miR-375 is decreased in T1D patients, indicating that the changed circulating level of miR-375 may be a valuable marker of inflammation and metabolic alterations in T1D [15]. The crosstalk between p38 mitogen-activated protein kinase signaling pathway and the lncRNA metastasis associated lung adenocarcinoma transcript 1 (*MALAT1*) is correlated with the endothelial cell function in diabetic rats, and *MALAT1* inhibition may be a promising approach for anti-angiogenic treatment of diabetic microvascular complications [16]. Besides, lncRNAs MEG3, TUG1 and PVT1 were also being reported to contribute in the pathophysiology of T1D and T1D-associated complications [17–19]. However, only a small part of non-coding RNAs in T1D have been revealed, and more comprehensive understanding of T1D should be developed to facilitate design of preventive therapeutic modalities.

MiiRNAs can silence gene expression by binding mRNAs, while competing endogenous RNAs (ceRNAs), such as lncRNAs can bind miRNAs via miRNA response elements competitively to regulate gene expression [20, 21]. In this study, the microarray dataset of T1D was searched from Gene Expression Omnibus (GEO) database. Afterwards, differential expression analysis, enrichment analysis, and lncRNA-miRNA-mRNA network (ceRNA network) analysis were executed to investigate the important regulatory axises involved in T1D.

## Methods

### Data source and data preprocessing

From GEO database (http://www.ncbi.nlm.nih.gov/geo/), the gene expression profile of T1D (accession number: GSE55100, including GPL570 and GPL8786 datasets) was downloaded. This dataset was deposited by Gu et al. [22]. There were 12 peripheral blood mononuclear cell (PBMC) samples from T1D patients and 10 PBMC samples from normal controls in both of GPL570 (including mRNA and lncRNA expression data) and GPL8786 (including miRNA expression data) datasets. The clinical characteristics of the 22 samples were listed in Table 1.

**Table 1:** Clinical characteristics of type 1 diabetes patients

	Control	T1D	*p*
Female/male	6/4	5/7	> 0.0.5
Age, years	18.70 ± 1.16	17.50 ± 3.68	> 0.05
FBG, mmol/L	4.78 ± 0.20	6.37 ± 1.93	< 0.05
HbA1C, %	5.29 ± 0.42	11.78 ± 3.63	< 0.001
GADA, U/mL	2.50 (1.87–2.91)	149.85 (47.30–319.33)	< 0.01
Fasting C peptide, ng/mL	2.08 ± 0.77	0.47 ± 0.20	< 0.001

Values are given as the mean ± SD or as median values with the interquartile range in parentheses. T1D, type 1 diabetes; FBG, fasting blood glucose; HbA1C, hemoglobin A1c; GADA, glutamic acid decarboxylase antibody

Using the Release 26 (GRCh38.p10) reference genome in GENCODE database (https://www.gencodegenes.org/human/) [23], sequence alignment was conducted. Only the unique alignment sequences were remained. Based on corresponding GTF annotation files, mRNAs (with annotation information of “protein coding”) and lncRNAs (with annotation information of “antisense”, “sense_intronic”, “lincRNA”, “sense_overlapping”, “processed_transcript”, “3prime_overlapping_ncRNA”, and “non_coding”) were identified, respectively. Subsequently, the probes without relevant gene symbols were filtered out. For multiple probes that mapped to one gene symbol, their average value was used as the final expression value of this gene.

### Differential expression analysis

For GPL570 and GPL8786 datasets, differential expression analysis between T1D and normal samples were performed using limma package (http://www.bioconductor.org/packages/2.9/bioc/html/limma.html) [24] in R. The corresponding *p* values of all genes were obtained through statistical test and were conducted with multiple test correction using Benjamini & Hochberg method [25]. The |log fold change (FC)|≥ 0.5 and adjusted *p* value < 0.05 were considered as the thresholds for obtaining differentially expressed mRNAs (DE-mRNAs). Meanwhile, the *p* value ≤ 0.05 was regarded as the cut-off criterion for selecting differentially expressed miRNAs (DE-miRNAs) and differentially expressed lncRNAs (DE-lncRNAs).

### Enrichment analysis for the DE-mRNAs

The Gene Ontology (GO)_biological process (BP), GO_molecular function (MF), and GO_cellular component (CC) functional terms involving the DE-mRNAs were analyzed using DAVID tool (version 6.7, https://david-d.ncifcrf.gov/) [26], and the corresponding results were visualized by the R package GOplot (http://cran.r-project.org/web/packages/GOplot) [27]. Using gene set enrichment analysis (GSEA) [28], Kyoto Encyclopedia of Genes and Genomes (KEGG) enrichment for the DE-mRNAs was carried out. The adjusted *p* value < 0.05 was used for screening the significant results.

### Construction of lncRNA-miRNA-mRNA interaction network

Using corr.test (parameters: ci = F, adjust = "BH") in R package psych [29], the Pearson correlation coefficients [30] of the expression values of the DE-lncRNAs and the DE-mRNAs were calculated. The lncRNA-mRNA pairs with |r|≥ 0.7 and adjusted *p* value < 0.05 were selected and a lncRNA-mRNA co-expression network was developed By using Cytoscape software (http://www.cytoscape.org) [31].

Using starbase database (version 3.0, http://starbase.sysu.edu.cn/) [32], lncRNA-miRNA interactions were predicted for the DE-lncRNAs in the lncRNA-mRNA co-expression network. Simultaneously, miRNA-mRNA interactions were predicted for the DE-mRNAs in the lncRNA-mRNA co-expression network by using with mirwalk database (version 3.0, http://mirwalk.umm.uni-heidelberg.de/) [33]. Finally, a lncRNA-miRNA-mRNA interaction network was visualized by Cytoscape software [31] by integrating the DE-lncRNAs and DE-mRNA regulated by the same DE-miRNA. Furthermore, GO and KEGG enrichment analysis for the mRNAs involved in the interaction network were conducted using DAVID tool [26].

### Validation of key genes using quantitatively real-time PCR (qRT-PCR)

T1D patients were recruited from the department of Endocrinology, China-Japan Union Hospital of Jilin University. Age and sex matched healthy controls were recruited from volunteers of physical examination. The experiments were approved by Ethics Committee of China-Japan Union Hospital of Jilin University [No.(2020)linshen(20,201,127)]. Written informed consent was received from all participants. Total RNA were extracted from the PBMCs of T1D patients and normal control samples using RNAiso Plus (TaKaRa, Shiga, Japan). The total RNA was reversed into cDNA with primeScript RT Master Mix (TaKaRa) and amplified on ABI ViiA7 (ThermoFisher, USA). GAPDH was used as the internal reference of DE-lncRNAs and DE-mRNAs and U6 was regarded as the internal reference of DE-miRNAs. The primers were listed in Table 2.

**Table 2:** The primers used for qRT-PCR

Primers	Sequences (5′–3′)
GAS5-hF	CACACAGGCATTAGACAGA
GAS5-hR	GCTCCACACAGTGTAGTCA
TRG-AS1-hF	CTCCTTCATTCCCTATTC
TRG-AS1-hR	TTATGATGGCTACGATGT
PPM1L-hF	GAGACCCGAGACGCTTTTCC
PPM1L-hR	GGCTGGACTTCACGATGGT
SERPINA1-hF	ATGCTGCCCAGAAGACAGATA
SERPINA1-hR	CTGAAGGCGAACTCAGCCA
hsa-miR-320a-3p-RT	GTCGTATCCAGTGCAGGGTCCGAGGTATTCGCACTGGATACGACGGGCGA
hsa-miR-320a-3p-F	GCGCGTCTCAACCCAGCTTTT
hsa-miR-23b-3p-RT	GTCGTATCCAGTGCAGGGTCCGAGGTATTCGCACTGGATACGACTACCAC
hsa-miR-23b-3p-F	GCGCATCCCTGGCAATGTGAT
hsa-miR-423-5p-RT	GTCGTATCCAGTGCAGGGTCCGAGGTATTCGCACTGGATACGACGACTTT
hsa-miR-423-5p-F	GCTCGCTCTCTGCCCCTCA
Universal primer	GTGCAGGGTCCGAGGT
hsa-U6-RT	GTCGTATCCAGTGCAGGGTCCGAGGTATTCGCACTGGATACGACAAAATATG
hsa-U6-F	CTCGCTTCGGCAGCACA
hsa-U6-R	AACGCTTCACGAATTTGCGT

### Statistical analysis

The data of qRT-PCR was processed by GraphPad Prism 5.0 (San Diego, CA, USA). Data were presented as mean ± standard deviation. Differences between two groups were determined by t-test. P < 0.05 was regarded as statistical significance level.

## Results

### Differential expression analysis

**Fig. 1:**
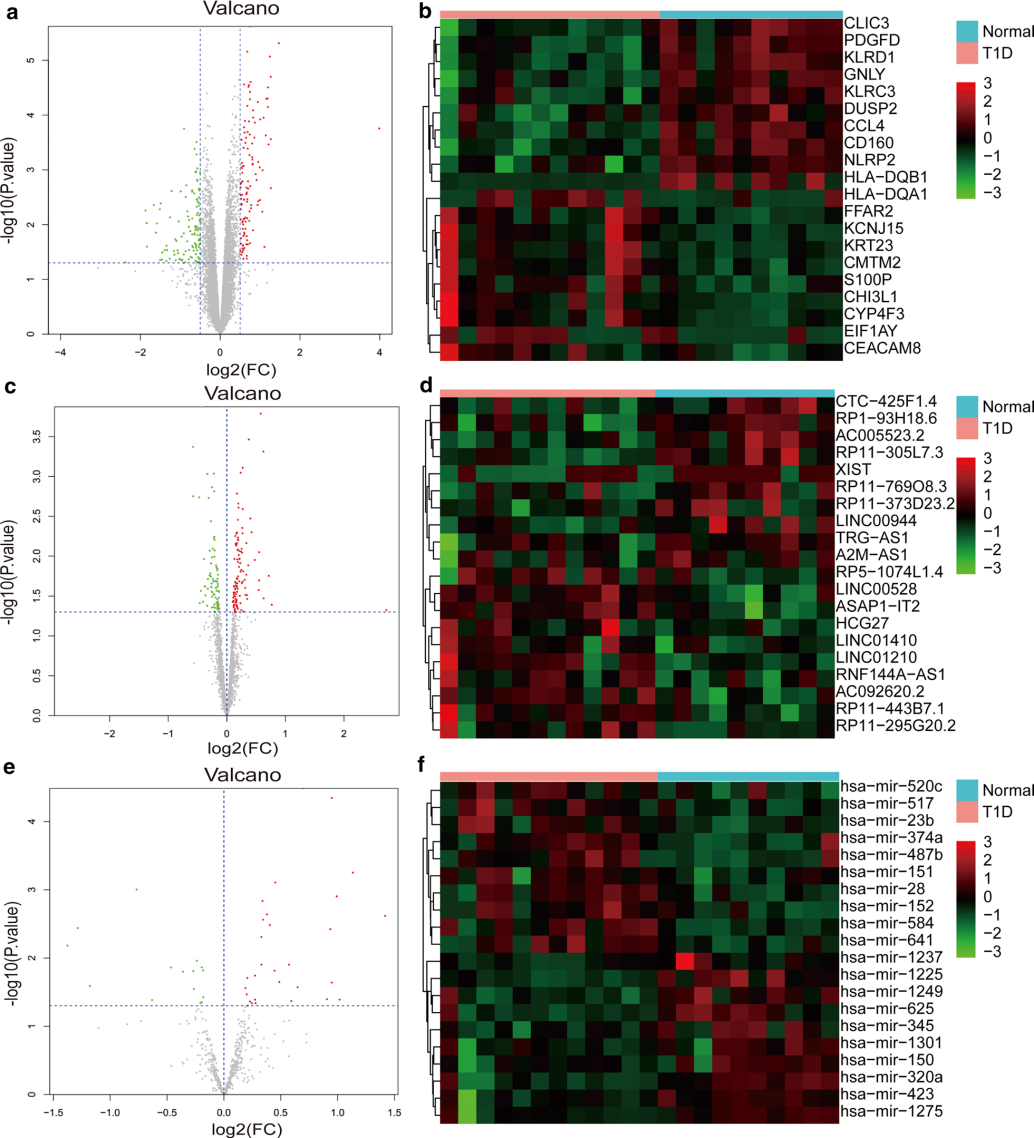
The results of differential expression analysis. **a** The volcano plot of the differentially expressed mRNAs (DE-mRNAs); **b** The expression heatmap of the top 10 up-regulated mRNAs and down-regulated mRNAs; **c** The volcano plot of the differentially expressed lncRNAs (DE-lncRNAs); **d** The expression heatmap of the top 10 up-regulated lncRNAs and down-regulated lncRNAs; **e** The volcano plot of the differentially expressed miRNAs (DE-miRNAs); **f** The expression heatmap of the top 10 up-regulated miRNAs and down-regulated miRNAs. FC, fold change. In volcano plots, red and green separately represent up-regulation and down-regulation. In expression heatmaps, red and blue separately represent type 1 diabetes (T1D) and normal samples

A total of 1671 mRNAs, 1990 lncRNAs, and 533 miRNAs were identified from the gene expression profile of GSE55100. Compared with normal samples, there were 236 DE-mRNAs (121 up-regulated and 115 down-regulated), 184 DE-lncRNAs (106 up-regulated and 78 down-regulated), and 45 DE-miRNAs (30 up-regulated and 15 down-regulated) in T1D samples (Fig. 1).

### Enrichment analysis for the DE-mRNAs

For the 236 DE-mRNAs, 16 GO_BP terms (such as “defense response”, adjusted *p* value = 5.01E-08; “immune response”, adjusted *p* value = 1.11E-04; and “response to wounding”, adjusted *p* value = 3.46E-04) and four GO_CC terms (such as “plasma membrane part”, adjusted *p* value = 2.72E-02; “intrinsic to plasma membrane”, adjusted *p* value = 2.90E-02; and “integral to plasma membrane”, adjusted *p* value = 3.46E-02) were enriched (Table 3).

**Table 3:** The Gene Ontology (GO) functional terms enriched for the differentially expressed mRNAs

Category	Term	Count	Genes	Adjusted *p* value
BP	GO:0006952 defense response	33	*CXCL1, PRF1, KLRC3, TNF, PGLYRP1, CXCR1, CCL5, CCL4, SH2D1A, IFNG, CSF3R, MS4A2, LTF, SERPINA1, CRISP3, IL18RAP, RNASE3, CAMP, GNLY, IL1RN, CHST2, CD160, SAMHD1, SLAMF7, CD180, NCR3, PROK2, TNFAIP6, KLRG1, CCR3, LYST, ALOX5, F2R*	5.01E-08
BP	GO:0006955 immune response	29	*HLA-DQB1, CXCL1, TNF, AQP9, PGLYRP1, CCL5, CCL4, CD96, SH2D1A, IFNG, CEACAM8, LTF, MS4A2, CRISP3, IL18RAP, GZMA, NCF4, IL1RN, EOMES, SAMHD1, SLAMF7, CD180, HLA-DQA1, CTSW, NCR3, CST7, ETS1, LYST, TGFBR3*	1.11E-04
BP	GO:0009611 response to wounding	24	*CXCL1, TNF, IL18RAP, IL1RN, CHST2, CXCR1, CCL5, CCL4, CD180, NCR3, PROK2, TNFAIP6, KLRG1, DYSF, ADM, CCR3, MTPN, MS4A2, JAK2, SERPINA1, ID3, ALOX5, NRG1, F2R*	3.46E-04
BP	GO:0006954 inflammatory response	18	*CXCL1, TNF, IL18RAP, IL1RN, CHST2, CXCR1, CCL5, CCL4, CD180, NCR3, TNFAIP6, PROK2, KLRG1, CCR3, MS4A2, SERPINA1, ALOX5, F2R*	6.96E-04
BP	GO:0006968 cellular defense response	8	*PRF1, SH2D1A, KLRC3, KLRG1, CCR3, GNLY, CD160, CCL5*	5.26E-03
BP	GO:0008285 negative regulation of cell proliferation	17	*CEBPA, COL18A1, CXCL1, TNF, PTPRM, ZBTB16, SKAP2, ADM, ETS1, IFNG, TGFBR3, JAK2, ADAMTS1, DLG5, IGFBP3, RUNX3, F2R*	7.51E-03
BP	GO:0009617 response to bacterium	12	*TNF, RNASE3, ADM, LYST, CAMP, GNLY, IFNG, PGLYRP1, LTF, SERPINA1, CCL5, F2R*	1.31E-02
BP	GO:0002703 regulation of leukocyte mediated immunity	7	*SH2D1A, TBX21, IFNG, MS4A2, CD226, LAG3, NCR3*	2.83E-02
BP	GO:0019835 cytolysis	5	*GZMM, PRF1, GZMA, GZMB, GZMH*	3.10E-02
BP	GO:0051270 regulation of cell motion	11	*COL18A1, PTPRM, ETS1, SMAD7, MMP9, RRAS2, TGFBR3, JAK2, ARAP3, IGFBP3, F2R*	3.59E-02
BP	GO:0031343 positive regulation of cell killing	5	*SH2D1A, IFNG, CD226, LAG3, NCR3*	3.87E-02
BP	GO:0030099 myeloid cell differentiation	8	*CEBPA, CDC42, TNF, MMP9, TGFBR3, JAK2, ZBTB16, FLVCR1*	3.92E-02
BP	GO:0042127 regulation of cell proliferation	24	*CEBPA, COL18A1, CXCL1, NAMPT, TNF, PTPRM, ZBTB16, SKAP2, HHEX, IL12RB1, ADM, ETS1, HLX, IFNG, TGFBR3, JAK2, ADAMTS1, DLG5, NRG1, TCF3, IGFBP3, RUNX3, F2R, MATK*	4.15E-02
BP	GO:0002697 regulation of immune effector process	8	*SH2D1A, TNF, TBX21, IFNG, MS4A2, CD226, LAG3, NCR3*	4.63E-02
BP	GO:0030334 regulation of cell migration	10	*COL18A1, PTPRM, SMAD7, MMP9, RRAS2, TGFBR3, JAK2, ARAP3, IGFBP3, F2R*	4.74E-02
BP	GO:0031341 regulation of cell killing	5	*SH2D1A, IFNG, CD226, LAG3, NCR3*	4.89E-02
CC	GO:0044459 plasma membrane part	48	*HLA-DQB1, KCNJ15, TNF, AQP9, APH1A, FFAR2, CD247, KCNJ2, SIRPB1, CD96, CDC42, IL12RB1, DYSF, CEACAM8, CSF3R, MS4A2, CSF2RB, DLG5, KLRF1, NRG1, KLRD1, LAG3, CSF2RA, FLVCR1, IL2RB, PTPRM, STX3, CD3G, SMAD7, GZMA, NCF4, SYT11, PTPN4, CD160, ABCB1, GZMB, HLA-DQA1, NCR3, TNFRSF10C, NPC1, CCR3, RRAS2, TGFBR3, JAK2, RGS9, NKG7, CD226, F2R*	2.72E-02
CC	GO:0031226 intrinsic to plasma membrane	31	*KCNJ15, TNF, AQP9, APH1A, FFAR2, KCNJ2, SIRPB1, CD96, IL12RB1, CEACAM8, CSF3R, MS4A2, CSF2RB, KLRF1, CSF2RA, FLVCR1, IL2RB, CD3G, PTPRM, SYT11, NCF4, CD160, HLA-DQA1, NCR3, NPC1, TNFRSF10C, CCR3, TGFBR3, NKG7, CD226, F2R*	2.90E-02
CC	GO:0005887 integral to plasma membrane	30	*KCNJ15, TNF, AQP9, APH1A, FFAR2, KCNJ2, SIRPB1, CD96, IL12RB1, CEACAM8, MS4A2, CSF2RB, CSF3R, KLRF1, CSF2RA, FLVCR1, IL2RB, CD3G, PTPRM, SYT11, NCF4, HLA-DQA1, NCR3, NPC1, TNFRSF10C, CCR3, TGFBR3, NKG7, CD226, F2R*	3.46E-02
CC	GO:0005886 plasma membrane	72	*KCNJ15, AQP9, CXCR1, SKAP2, LGR6, SIRPB1, CDC42, CD96, DYSF, CEACAM8, OSCAR, S1PR5, SMAGP, CSF2RB, MS4A2, CSF3R, DLG5, NRG1, KLRD1, CSF2RA, LAG3, FLVCR1, KLRB1, STX3, CD3G, PTPRM, GZMA, GPR171, NCF4, CD160, SIGLEC10, GZMB, BASP1, HLA-DQA1, NCR3, NPC1, TNFRSF10C, CCR3, RRAS2, PTGDR, CD226, HLA-DQB1, ENPP5, PRF1, GPRC5D, TNF, APH1A, FFAR2, CD247, ZBTB16, KCNJ2, ACSL1, IL12RB1, COL6A3, KLRF1, IL2RB, SMAD7, SYT11, PTPN4, ABCB1, CD180, TIGIT, P2RY13, TGFBR3, JAK2, RGS9, ALOX5, ARAP3, NKG7, GCA, F2R, FEZ1*	3.88E-02

BP, biological process; CC, cellular component

Meanwhile, 57 significant pathways were enriched for the DE-mRNAs, including 23 activated pathways (normalized enrichment score (NES) > 0; such as “Th1 and th2 cell differentiation”, adjusted *p* value = 6.56E-03; “T cell receptor signaling pathway”, adjusted *p* value = 6.56E-03; and “Biosynthesis of unsaturated fatty acids”, adjusted *p* value = 6.56E-03) and 34 suppressed pathways (NES < 0; such as “Leishmaniasis”, adjusted *p* value = 6.56E-03; “Legionellosis”, adjusted *p* value = 6.56E-03; and “Phagosome”, adjusted *p* value = 6.56E-03) (Table 4).

**Table 4:** The activated pathways and suppressed pathways enriched for the differentially expressed mRNAs (top 10 listed)

Description	Normalized enrichment score	Genes	Adjusted *p* value
*Up-regulated pathways*			
Natural killer cell mediated cytotoxicity	1.88803	*KLRC3, KLRD1, IFNG, GZMB, PRF1, NCR3, SH2D1A, CD247, NCR1, FASLG, PIK3R3, PPP3CC, CD244, HCST, ZAP70, KLRC4, FYN, KIR2DL1, PIK3R1, PLCG1, LCK, KIR3DL1, LAT*	6.56E-03
Biosynthesis of unsaturated fatty acids	1.989893	*ELOVL6, ACOT7, TECR/ACOT4, SCD5, FADS2*	6.56E-03
T cell receptor signaling pathway	2.006524	*IFNG, CD3G, CDC42, CD247, PTPRC, CD8A, PIK3R3, PPP3CC, MAPK13, CD3D, PRKCQ, AKT3, ITK, ZAP70, RASGRP1, FYN, GRAP2, PIK3R1, PDPK1, PLCG1, NFATC3, MAPK9, LCK, LAT, NCK2*	6.56E-03
TH1 and TH2 cell differentiation	2.137425	*IFNG, CD3G, RUNX3, IL2RB, TBX21, STAT4, IL12RB1, CD247, PPP3CC, MAPK13, CD3D, PRKCQ, ZAP70, GATA3, PLCG1, NFATC3, IL12RB2, IL2RG, MAPK9, LCK, LAT, DLL1, MAML3*	6.56E-03
RNA degradation	1.717539	*PFKP, MPHOSPH6, PAPD5, HSPA9, DDX6, EXOSC2, LSM2, LSM5, TOB1, DCP1B, LSM1, TOB2, EXOSC7, LSM4, ENO2, DHX36, LSM8, CNOT3, PFKM, ZCCHC7, BTG1, LSM6, EXOSC3, LSM7, EXOSC1, PNPT1, EXOSC5, LSM3, DCPS*	8.48E-03
Ribosome biogenesis in eukaryotes	1.778302	*LSG1, WDR36, AK6, WDR43, RIOK1, POP5, EMG1, RPP40, RAN, EIF6, NXT2, POP7, SPATA5, RBM28, GNL3L, NVL, GAR1, NHP2, BMS1, DROSHA, GNL2, FBL, XPO1, POP1/RCL1, NXT1, IMP4, UTP18, POP4, WDR3, CSNK2A2, NAT10*	8.48E-03
TH17 cell differentiation	1.854181	*IFNG, RORA, CD3G, IL2RB, TBX21, IL12RB1, CD247, PPP3CC, MAPK13, CD3D, PRKCQ, ZAP70, GATA3, PLCG1, NFATC3, IL2RG, MAPK9, LCK, LAT, IL21R*	8.48E-03
Proteasome	1.88607	*IFNG, PSMD11, PSMD14, PSMB9, PSMD6, PSMA3, PSMB2, PSMA1, PSMA5, PSMA7, PSMB10, PSMB4, PSME1, PSMB6, PSMC2, PSMB5, PSMD2, PSMD8, PSME4, PSMC5, POMP, PSMD12, PSMA2, PSMB1*	8.48E-03
Graft versus host disease	1.87728	*KLRD1, IFNG, GZMB, PRF1, FASLG, KIR2DL1*	8.97E-03
Fatty acid elongation	1.857462	*ELOVL6, ACOT7, TECR, ACOT4, ELOVL7, ACAA2*	1.14E-02
*Down-regulated pathways*			
Leishmaniasis	− 1.90122	*MYD88, PTPN6, JAK1, ITGB1, CYBB, IL1A, TLR2, IRAK1, NCF2, TAB2, NCF1, IFNGR2, CR1, NCF4, JAK2, TNF, PTGS2, FCGR3B*	6.56E-03
Legionellosis	− 1.89969	*NLRC4, CD14, CASP1, EEF1A2, TLR2, IL18, EEF1A1, HSPD1, APAF1, CR1, CXCL8, TNF, HSPA6, CXCL1*	6.56E-03
Phagosome	− 1.86804	*TUBA1C, TUBA1A, THBS1, C3, CORO1A, ATP6V1C1, RAB5C, ITGB1, HGS, TUBB1, ATP6V1B2, CD36, THBS3, ATP6V0B, FCGR2B, MRC1, TUBB2A, CD14, ATP6V0C, THBS4, CYBB, RAB7A, TLR2, CLEC7A, ATP6V1A, ATP6V0D1, NCF2, TLR6, NCF1, FCAR, LAMP1, CTSS, NCF4, OLR1, FCGR3B, MPO*	6.56E-03
Hematopoietic cell lineage	− 1.7603	*FCER2, CD1D, CD22, CD1C, CD4, CD37, CR2, CD19, CSF1R, IL3RA, CD36, IL11RA, CD14, IL9R, IL1A, ANPEP, MS4A1, CD9, CR1, IL1R1, CSF2RA, CD59, MME, TNF, CSF3R, IL1R2, CD24*	6.56E-03
Tuberculosis	− 1.74971	*RAB5A, MAPK10, TLR9, RFXAP, RAB5B, FCER1G, IFNA7, SPHK2, CLEC4E, SYK, RAF1, ATP6AP1, CALML5, MAPK8, C3, FADD, MYD88, CEBPB, CORO1A, JAK1, RAB5C, CREB1, IL10RB, KSR1, LSP1, NOD2, ATP6V0B, FCGR2B, SPHK1, BCL10, MRC1, NFYC, IFNA17, CD14, CAMK2G, ATP6V0C, RAB7A, IL1A, TLR2, IL18, CLEC7A, EP300, TLR1, ATP6V0D1, IRAK1, CTSD, TLR6, HSPD1, IFNGR2, RHOA, LAMP1, APAF1, CR1, CTSS, JAK2, TNF, FCGR3B, CAMP*	6.56E-03
Protein digestion and absorption	− 1.70571	*COL27A1, SLC6A19, SLC8A1, SLC7A7, KCNQ1, CELA3B, PRSS3, COL5A3, COL1A2, ATP1B1, COL9A3, SLC7A8, COL9A2, COL17A1, KCNE3, COL6A3, COL18A1, MME, CPA3*	6.56E-03
Cytokine cytokine receptor interaction	− 1.48441	*CCR10, IL3RA, IL17RA, TNFSF13, IL19, CCL8, EGFR, IL11RA, OSM, CXCL12, CXCR5, IFNLR1, TNFRSF18, IL17RC, PRL, IFNA17, PLEKHO2, IL9R, IL17RB, IL1RAP, IL1A, IL18, HGF, VEGFA, CCR6, IFNGR2, TNFSF10, PF4V1, CXCL8, CCL20, TNFSF8, IL1R1, CSF2RA, CCL2, CCR9, CSF2RB, TNF, TNFRSF10C, CXCR2, CXCR1, CSF3R, CXCL1, IL1R2, TNFRSF17, CCR3*	6.56E-03
Starch and sucrose metabolism	− 1.89049	*GBE1, G6PC2, GPI, GAA, HK3, G6PC3, PGM2L1, ENPP3, PYGL, MGAM*	8.48E-03
Glycine serine and threonine metabolism	− 1.80927	*GAMT, PGAM2, AOC3, BPGM, AOC2, ALAS1, GLDC, ALAS2*	8.48E-03
Amoebiasis	− 1.70409	*PRKACA, RAB5C, SERPINB10, LAMB2, LAMB3, GNAQ, COL1A2, CD14, PLCB2, RAB7A, TLR, LAMA5, ACTN1, CXCL8, IL1R1, TNF, CTSG, CXCL1, IL1R2, ARG1*	8.48E-03

### Construction of lncRNA-miRNA-mRNA interaction network

**Fig. 2:**
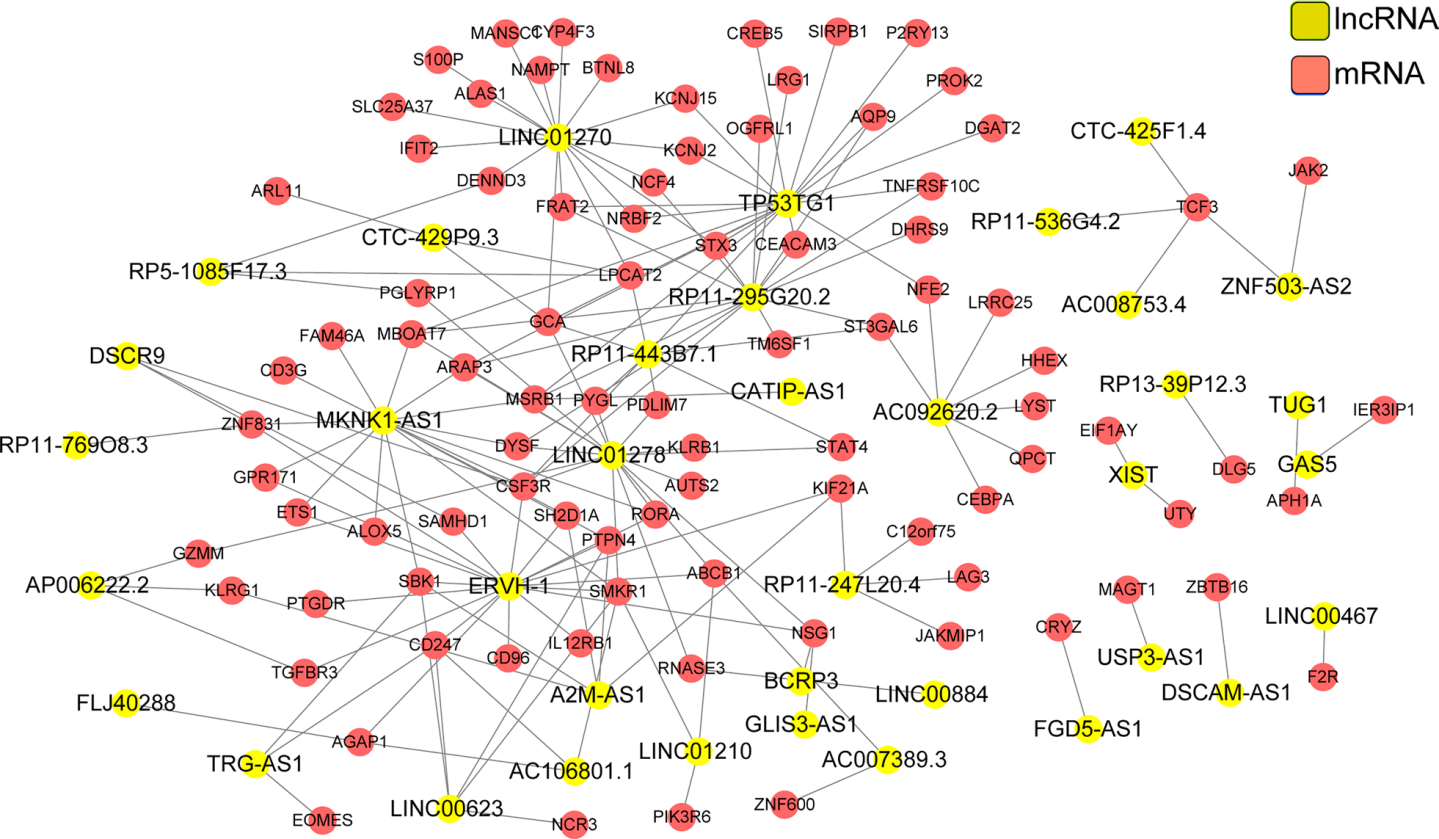
The long non-coding RNA (lncRNA)-mRNA co-expression network. Red and yellow circles separately represent mRNAs and lncRNAs

**Fig. 3:**
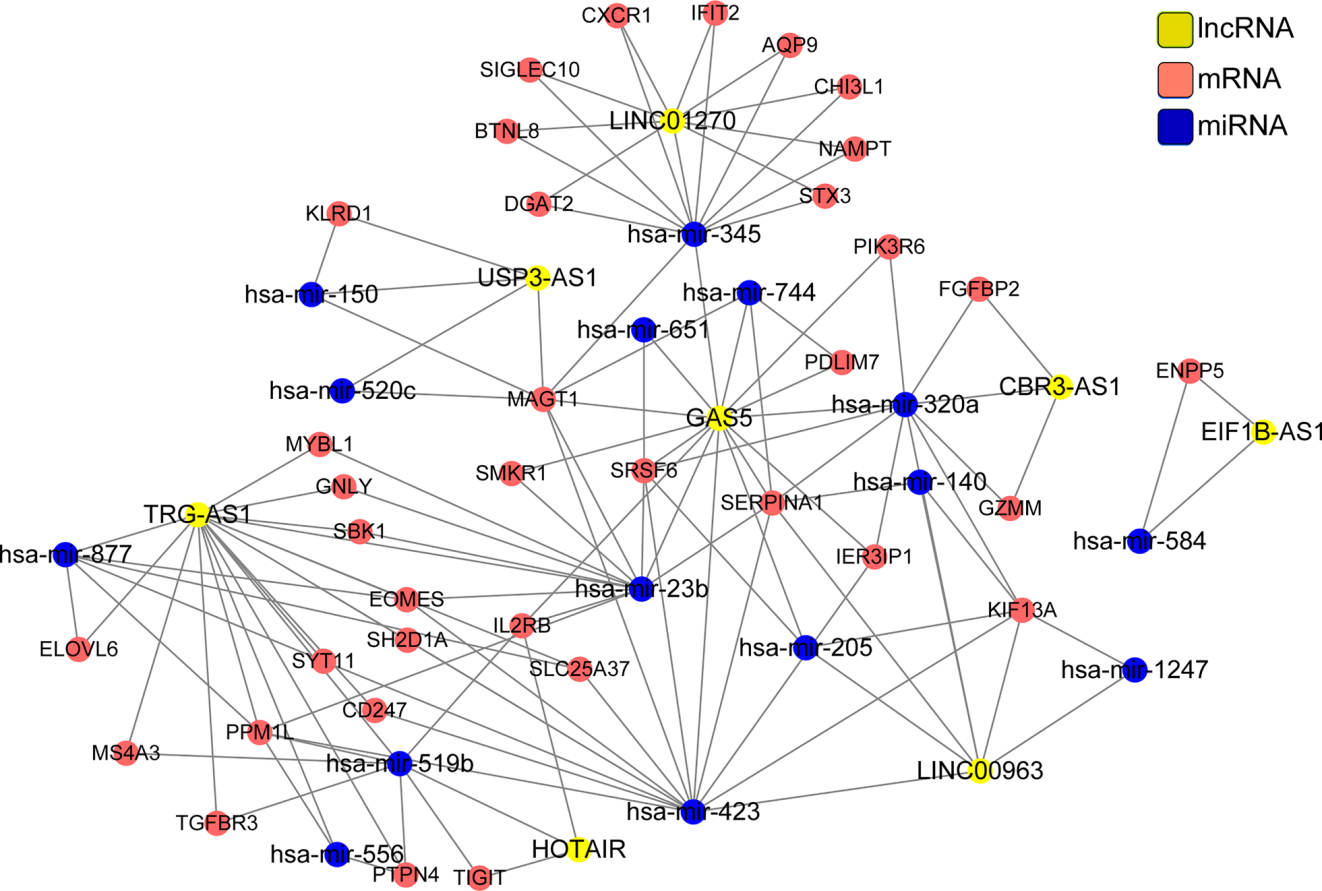
The long non-coding RNA (lncRNA)-miRNA-mRNA interaction network. Red, yellow, and blue circles represent mRNAs, lncRNAs, and miRNAs, respectively

In the lncRNA-mRNA co-expression network, there were 127 nodes (37 lncRNAs and 90 mRNAs) and 174 edges (Fig. 2). After integrating the results of lncRNA-miRNA interaction prediction and miRNA-mRNA interaction prediction, the lncRNA-miRNA-mRNA interaction network (including 36 mRNAs, 8 lncRNAs, and 15 miRNAs) was constructed (Fig. 3). Importantly, T cell receptor gamma locus antisense RNA 1 (*TRG-AS1*)—miR-23b/miR-423—protein phosphatase, Mg2 + /Mn2 + dependent 1L (*PPM1L*) and growth arrest specific 5 (*GAS5*) —miR-320a/miR-23b/miR-423—serpin family A member 1 (*SERPINA1*) regulatory axises were implicated in the interaction network.

For the mRNAs involved in the interaction network, two GO_BP terms (“Adaptive immune response”, *p* value = 3.41E-02; “Regulation of immune response”, *p* value = 4.76E-02), three GO_CC terms (“Endoplasmic reticulum”, *p* value = 1.78E-02; “Integral component of membrane”, *p* value = 3.56E-02; “ER to Golgi transport vesicle”, *p* value = 4.74E-02), one GO_MF term (“Protein homodimerization activity”, *p* value = 4.78E-02), and one KEGG pathway (“Natural killer cell mediated cytotoxicity”, *p* value = 1.83E-02) were enriched (Table 5).

**Table 5:** The Gene Ontology (GO) functional terms and pathways enriched for the mRNAs involved in the long non-coding RNA (lncRNA)-miRNA-mRNA interaction network

Category	ID	Description	*p* value
BP	GO:0002250	Adaptive immune response	3.41E-02
BP	GO:0050776	Regulation of immune response	4.76E-02
CC	GO:0005783	Endoplasmic reticulum	1.78E-02
CC	GO:0016021	Integral component of membrane	3.56E-02
CC	GO:0030134	ER to Golgi transport vesicle	4.74E-02
MF	GO:0042803	Protein homodimerization activity	4.78E-02
Pathway	hsa04650	Natural killer cell mediated cytotoxicity	1.83E-02

BP, biological process; CC, cellular component; MF, molecular function

### Validation of key genes using qRT-PCR

**Fig. 4:**
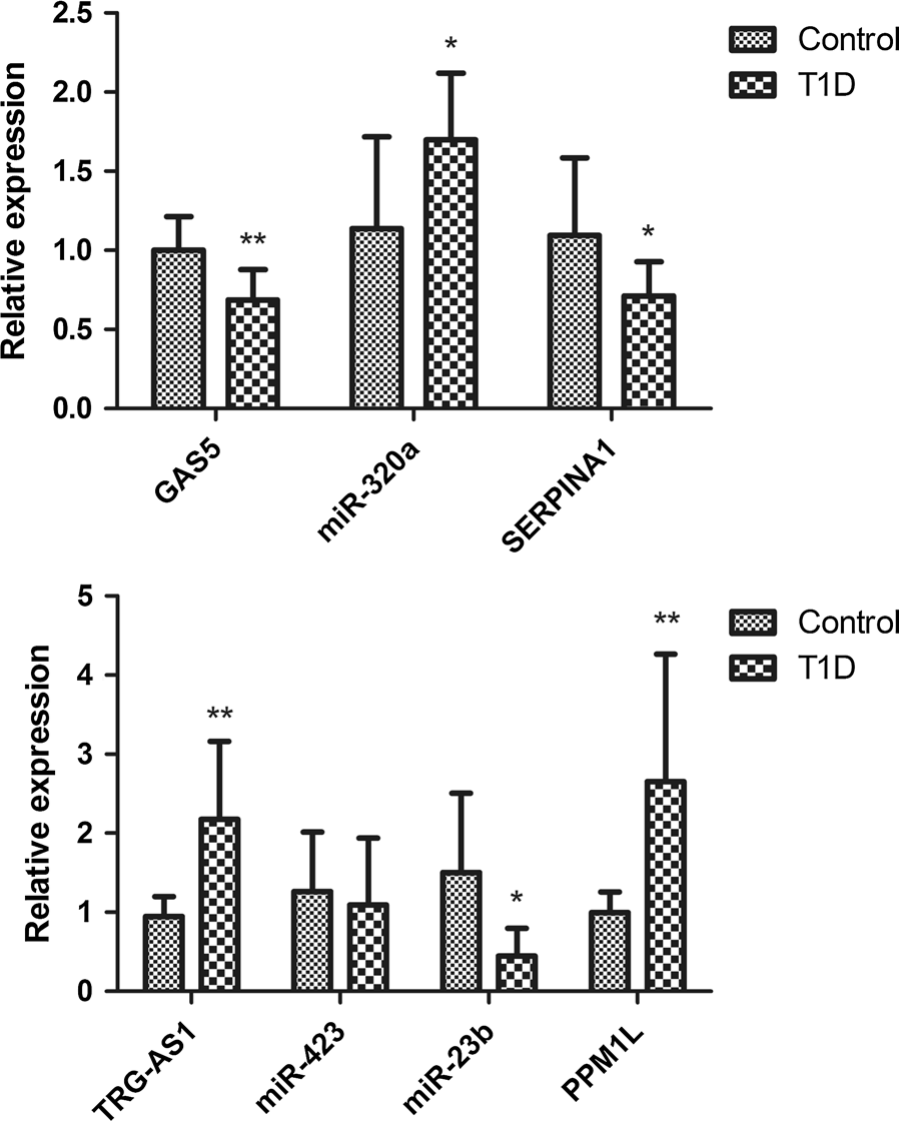
Validation of key genes using quantitatively real-time PCR. *p < 0.05, **p < 0.01 compared with the control group

In order to verify the results of bioinformation analysis, the expression of *TRG-AS1*-miR-23b/miR-423-*PPM1L* and *GAS5*-miR-320a /miR-23b/ miR-423- *SERPINA1* were verified in T1D patients and normal controls. According to the differential expression analysis, *TRG-AS1*, miR-320a and *PPM1L* were upregulated in T1D patients, while *GAS1*, miR-423, miR-23b and *SERPINA1* were downregulated in T1D patients. As shown in Fig. 4, the expression levels of *TRG-AS1*- miR-23b -*PPM1L* and *GAS5*-miR-320a- *SERPINA1* were consistent with bioinformation analysis (*p* < 0.05). No significant difference was detected in miR-423 between control group and T1D group (*p* > 0.05).

## Discussion

In recent years, the critical roles of non-coding RNAs including lncRNAs and miRNAs in diabetes were recognized. However, little is known about the molecular mechanism of them in regulating the development of T1D. In this study, 236 DE-mRNAs (121 up-regulated and 115 down-regulated), 184 DE-lncRNAs (106 up-regulated and 78 down-regulated), and 45 DE-miRNAs (30 up-regulated and 15 down-regulated) between T1D samples and normal samples were selected. For the 236 DE-mRNAs, 16 GO_BP terms, four GO_CC terms, and 57 significant pathways were enriched. Moreover, by constructing an lncRNA-miRNA-mRNA co-expression network, two interactions, *TRG-AS1*—miR-23b/ miR-423—*PPM1L* and *GAS5*—miR-320a/miR-23b/miR-423—*SERPINA1* regulatory axises were identified.

TRG-AS1 has been reported to be oncogenic in glioblastoma [34], hepatocellular carcinoma [35] and tongue squamous cell carcinoma [36]. It might act as a ceRNA to regulate miRNA-543-Yes-associated protein 1 in tongue squamous cell carcinoma [36], miR-4500-BACH1 in hepatocellular carcinoma [35] and miR-877-5p-SUZ12 in glioblastoma [34]. It also identified to be involved in repeated implantation failure [37]. However, its role in diabetes was not characterized previously. In our study, we predicted the miRNAs and mRNAs that might be regulated by TRG-AS1. The lncRNA-miRNA-mRNA network displayed that it could directly regulate 5 miRNAs including miR-423 and miR-23b, and multiple mRNAs, including *PPM1L* indirectly. MiR-23b/27b expression is decreased in the muscle stem cells of T2D patients, which exerts a pro-myogenic function via the p53 pathway [38]. Nuclear factor, erythroid 2/miR-423-5p axis can induce gluconeogenesis and hyperglycemia through inhibiting the family with sequence similarity 3 member A -adenosine triphosphate (ATP)-serine/threonine kinase Akt pathway, which is involved in the progression of T2D and nonalcoholic fatty liver disease [39]. Increased miR-320 impairs lipid metabolism and gluconeogenesis by targeting adiponectin receptor 1 (*AdipoR1*), and thus miR-320 may be taken as a possible target for T2D therapy [40]. The elevated levels of the T lymphocytes expressing gamma-delta T cell receptor are implicated in the islet autoimmune process in individuals at high risk of T1D, which may serve as a promising indicator for the development of T1D [41, 42]. *PPM1L* mediates inositol-requiring protein-1 phosphorylation and endoplasmic reticulum stress signaling, which is considered as a causal gene for metabolic abnormalities [43]. The macrophage-enriched network has a causal correlation with metabolic disease traits, which involved three obesity genes (including *PPM1L*) [44]. Therefore, we hypothesized that *TRG-AS1* and *PPM1L* might also play roles in the mechanisms of T1D through the *TRG-AS1*—miR-23b/miR-423—*PPM1L* regulatory axis.

GAS5 is a member of 5′ terminal oligopyrimidine class which could regulate cell growth, proliferation, and survival [45]. Reduced serum levels of *GAS5* are related to diabetes; therefore, serum *GAS5* levels combined with other parameters may be used for identifying people at high risk of diabetes more accurately [46]. Shi et al. demonstrated that GAS5 regulates insulin signaling in adipocytes, and suggested it might be a potential target for T2DM [47]. *GAS5* knockdown causes cell cycle arrest and impairs insulin synthesis and secretion in Min6 pancreatic β-cells, and thus *GAS5* may function in maintaining the identity and function of β cells [48]. In this study, GAS5 was found decreased in T1D patients in both RNA-seq results and the qRT-PCR results, which is consistent with previous studies. We found GAS5 could regulate 7 miRNAs, including miR-320a, miR-23b and miR-423, and tens of mRNAs, including *SERPINA1* in T1D. MiR-320 negatively mediates the expression of fibronectin, endothelin 1, and vascular endothelial growth factor via extracellular signal-regulated kinases 1 and 2 in high glucose-treated human umbilical vein endothelial cells, which may provide a novel approach for treating diabetic complications [49]. Wei et al. found miR-320 mimic correlated with impaired gluconeogenesis and lipid metabolism by regulating adipoR1 [40]. SERPINA1 is a serine protease inhibitor that could target elastase, plasmin, thrombin, trypsin, chymotrypsin, and plasminogen activator. It was shown to be decreased in serum of obese mice and human subjects and the imbalance between SERPINA1 and neutrophil elastase contributed to insulin resistance [50]. Lower levels of SERPINA1 selectively impaired the ATP-binding cassette transporter A1 cholesterol efflux capacity in T2D [51]. Other members in this family were also reported to participate in development of diabetes. Anti-SERPINB13 antibody contributes to Reg gene expression and beta cell proliferation, and the immunological response may hinder the progression of T1D [52]. The serum concentrations of *SERPINA12* (vaspin) is increased in T2D patients, which may be a candidate marker for evaluating the risk of severe macrovascular complications and the status of old T2D patients [53]. Thus, *GAS5*—miR-320a/miR-23b/miR-423—*SERPINA1* regulatory axis might also function in the pathogenesis of T1D.

## Conclusion

In conclusion, 236 DE-mRNAs, 184 DE-lncRNAs, and 45 DE-miRNAs between T1D and normal samples were identified. Besides, *TRG-AS1*—miR-23b/miR-423—*PPM1L* and *GAS5*—miR-320a/miR-23b/miR-423—*SERPINA1* regulatory axises might be related to the pathogenesis of T1D. Though we have validated the expression of these genes in T1D patients, further mechanically mechanism of these regulatory axises should be investigated by subsequent studies.

## Data Availability

The dataset analysed during the current study are available in the GEO repository with accession number of GSE55110 (https://www.ncbi.nlm.nih.gov/geo/query/acc.cgi?acc=GSE55100), GPL570 (https://www.ncbi.nlm.nih.gov/geo/query/acc.cgi?acc=GPL570), GPL8786 (https://www.ncbi.nlm.nih.gov/geo/query/acc.cgi?acc=GPL8786). The reference genome of Release 26 (GRCh38.p10) is available in GENCODE database (https://www.gencodegenes.org/human/). The interactions between lncRNA and miRNAs were obtained from starbase (http://starbase.sysu.edu.cn/). The miRNA-mRNA interactions were obtained from mirwalk database (http://mirwalk.umm.uni-heidelberg.de/).

## Abbreviations

T1D: Type 1 diabetes
lncRNA: Long non-coding RNA
miRNA: MicroRNA
DEmRNA: Differentially expressed mRNAs
GO: Gene ontology
BP: Biological process
CC: Cellular component
T2D: Type 2 diabetes
Tregs: Regulatory T cells
NFAT5: Nuclear factor of activated T cells 5
MALAT1: Metastasis Associated Lung Adenocarcinoma Transcript 1
ceRNAs: Competing endogenous RNAs
GEO: Gene Expression Omnibus
PBMC: Peripheral blood mononuclear cell
FC: Fold change
GSEA: Gene set enrichment analysis
KEGG: Kyoto Encyclopedia of Genes and Genomes
qRT-PCR: Quantitatively real-time PCR
NES: Normalized enrichment score
TRG-AS1: T cell receptor gamma locus antisense RNA 1
PPM1L: Protein Phosphatase: Mg2 + /Mn2 + Dependent 1 l
GAS5: Growth arrest specific 5
SERPINA1: Serpin family A member 1
ATP: Adenosine triphosphate
AdipoR1: Adiponectin Receptor 1
